# Increased Prevalence of *Salmonella* Infantis Isolated from Raw Chicken and Turkey Products in the United States Is Due to a Single Clonal Lineage Carrying the pESI Plasmid

**DOI:** 10.3390/microorganisms10071478

**Published:** 2022-07-21

**Authors:** Elizabeth A. McMillan, Margaret D. Weinroth, Jonathan G. Frye

**Affiliations:** 1 Poultry Microbiological Safety and Processing Research Unit, United States Department of Agriculture, Agricultural Research Service, U.S. National Poultry Research Center, Athens, GA 30605, USA; elizabeth.mcmillan@usda.gov (E.A.M.); maggie.weinroth@usda.gov (M.D.W.); 2 Bacterial Epidemiology Antibiotic Resistance Research Unit, United States Department of Agriculture, Agricultural Research Service, U.S. National Poultry Research Center, Athens, GA 30605, USA

**Keywords:** *Salmonella* Infantis, pESI plasmid, poultry

## Abstract

Infantis has recently become one of the most common serotypes of *Salmonella* isolated in the U.S. from raw meat samples collected in processing facilities and in retail stores. Investigations have determined that the majority of these isolates contain the pESI plasmid, but there has not been a large-scale investigation of the chromosome of these isolates. Here, we investigated 3276 whole-genome sequences of *Salmonella* Infantis with and without the pESI plasmid to understand chromosomal differences between plasmid carriage groups. *S*. Infantis genomes arranged into multiple clades with a single clade containing the isolates carrying the plasmid. Fifty-eight SNPs were identified in complete linkage disequilibrium between isolates that did and did not carry the plasmid. However, there were no unique genes present only in the genomes of isolates containing the plasmid. On average, isolates with the plasmid did contain more insertion sequences than those without (*p* < 0.05). Given that *S*. Infantis isolates carrying pESI form a single clade, it can be inferred that the increase in carriage of this plasmid in the U.S. is due to rapid clonal expansion of a single strain rather than as a result of multiple transfer events. As this *S*. Infantis clone does not contain any unique chromosomal genes, its proliferation appears to be due to pESI plasmid-encoded genes that may be advantageous in the chickens and turkeys or in their environment.

## 1. Introduction

*Salmonella enterica* is a leading cause of foodborne illness in the United States, with many of these illnesses being attributed to poultry sources [1]. Although there are more than 2500 serotypes of *Salmonella*, the vast majority of human illnesses are caused by those serotypes from subspecies I [2]. Since 2016, Infantis has emerged as one of the most commonly isolated serotypes of *Salmonella* from poultry sources in the U. S., and human infections due to serotype Infantis have increased as well [1,3,4]. Unfortunately, many of these *Salmonella* Infantis infections have exhibited resistance to cephalosporins, ciprofloxacin, and other antibiotics, limiting the options for treatment [1]. 

In 2018, the Centers for Disease Control and Prevention (CDC) initiated an outbreak investigation for a strain of *S.* Infantis associated with raw chicken products. The investigation closed in 2019 with the determination that there was no point source for the strain [5]. As a part of the investigation, it was determined that this outbreak strain contained the pESI plasmid. First described in Israel in 2014, pESI has been detected in *S.* Infantis isolates from chickens, food samples, and human clinical cases in South America, Europe, Africa, and Asia [6,7,8]. It has since been determined that the majority of *S*. Infantis isolates collected from raw chicken and turkey in the U.S. carry pESI and that the majority of these plasmids contain genes that could confer an advantage in a poultry host. Advantageous genes include unique fimbriae, a siderophore iron transport system, heavy-metal resistance genes, and antibiotic resistance genes [9]. Of particular concern is the *bla*_CTX-M-65_ gene, which conveys extended-spectrum β-lactam resistance and is present in approximately half of pESI plasmids in the U.S. [9].

A 2021 study by Tyson et al. showed that several thousand publicly available *S*. Infantis whole-genome sequences containing pESI could be grouped together by less than 50 single-nucleotide polymorphism (SNP) differences in the Isolates Browser of the Pathogen Detection database hosted by the NCBI [10]. Further, 31 isolates carrying pESI were subjected to long-read sequencing and found to be highly related. A study of 100 *S*. Infantis genomes collected globally also found that isolates carrying the pESI plasmid belonged to their own clade [11]. Although these studies and others have hypothesized and concluded that carriage of the pESI plasmid has influenced the expansion of *S*. Infantis in U.S. poultry, there has not been a large-scale investigation of the *S*. Infantis chromosome in U.S. isolates. In the present study, we investigated more than 3000 *S*. Infantis genomes sequenced in the U.S. from carcass rinses collected at processing, retail meat, human infections, and imported food samples to define the *S*. Infantis strains carrying pESI and to investigate the role of the chromosome in the proliferation of *S*. Infantis in U.S. poultry.

## 2. Materials and Methods

All *Salmonella enterica* whole-genome assemblies uploaded to the Pathogen Detection Isolates Browser hosted by the NCBI with the following parameters were downloaded on 11/30/2021 (*n* = 4433): serovar Infantis (serovar assigned by submitter at upload), and collected by the CDC, United States Food and Drug Administration (FDA), or United States Food Safety and Inspection Service (USDA-FSIS). Genome contigs were aligned to ten target sequences as described previously (*ard*A, *tra*I, IncP, *ipf*, *mer*A, pESI *rep*A, *pil*L, *sog*S, *trb*A, and YTB [9]) to determine if the plasmid was present using BWA-MEM (version 0.7.17) and coverage was calculated with the pileup script from bbtools (version 38.79) (https://sourceforge.net/projects/bbmap/, accessed 1 February 2020) [12]. The plasmid was considered present if all targets were present at 100% coverage. 

Core SNPs were found using parsnp (version 1.2); from this preliminary VCF file, possible non-Infantis serotypes and outgroups were identified and further investigated through BLAST searches or parsnp log cluster coverage, respectively [13]. Those found to be associated with an outgroup (>19,000 SNP difference from reference) or the incorrect serotype were removed. From there, all remaining strains were included in a core genome analysis using parsnp with FSIS150296 (ASM193157v1) used as the reference strain and the “-c” parameter to force inclusion of all genomes. Output was converted to a VCF using harvest tools [13]. Linkage disequilibrium (LD) was determined visually by inspection of the VCF file, with those SNPs in 100% LD being reported (meaning if all strains that did or did not carry a plasmid differed in the same way across all genomes, they were considered in 100% LD). The VCF files were evaluated using R (4.0.2) to identify single-nucleotide polymorphisms (SNPs) that varied between strains that did and did not carry the plasmid. The reference Genbank file was used to identify which genes SNPs of interest occurred within a higher level of gene ontology assigned by BlastKOALA [14]. IQ-TREE (version 2.0-rc1) was used for construction of a phylogenetic tree with default settings and visualized and tabled with Fig Tree (version 1.4.4) (https://github.com/rambaut/figtree/, accessed after 25 November 2018) [15]. 

Prokka (version 1.14.5) was used to annotate all strains [16]. A subset of closed genomes with plasmid carriage as well as four closed plasmids were annotated to identify which genes were associated with the plasmid so they could be removed downstream. Roary (version 3.12.0), with the default settings, was used to generate a pan-genome [17]. Scoary (version 1.6.16) was used to calculate the number of genes present in plasmid carriage and non-carriage groups. Two different criteria were used to make this calculation: strict (gene occurring in ≥99% of one classification and ≤1% of the other) and relaxed (gene occurring in ≥95% and ≤5%) [18]. 

ISEscan (version 1.7.2.2) was used for identification of insertion elements using default settings with contigs associated with the chromosome used as input [19]. A subset of publicly available closed plasmids was also subjected to ISEscan and any IS element found within these plasmids was removed from IS element counts for the non-closed genomes. Differences in IS element numbers between strains that carried and did not carry the plasmid were assessed with the ANOVA function in the car program in R using the following packages: ggplot2 (version 3.3.5), rworldmap (version 1.3-6), and usmap (version 0.6.0).

## 3. Results

### 3.1. Curating of Publicly Available Strains from NCBI

**Figure 1:**
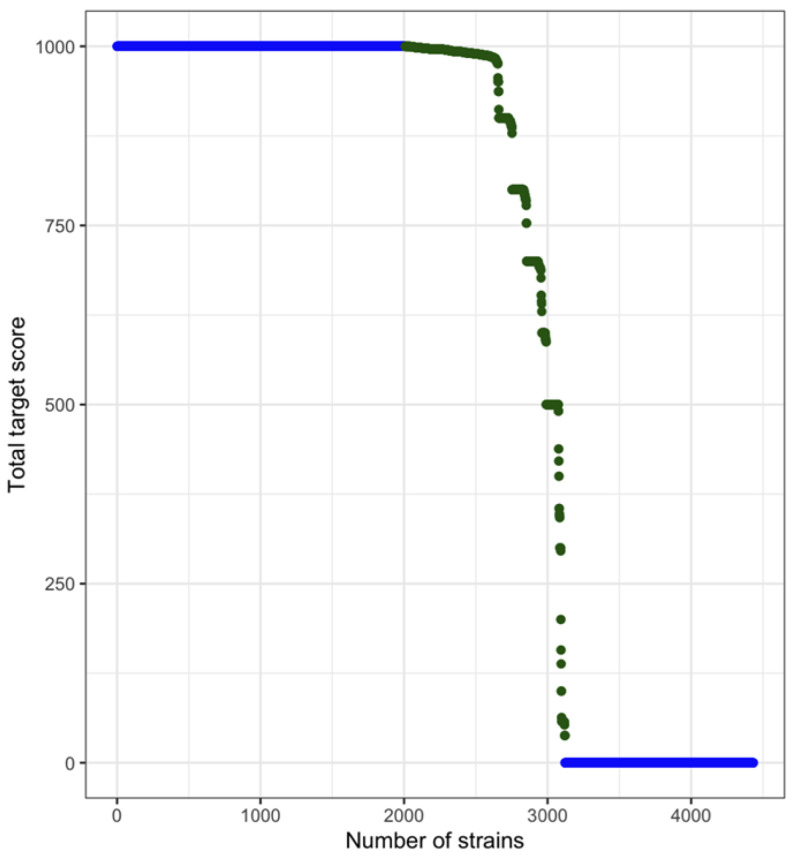
Overview of number of strains of *Salmonella* Infantis that harbored targets associated with the pESI plasmid; all ten targets at a coverage of 100% resulted in a score of 1000 and classified the plasmid as present. Strains that had no coverage of a target (0) were classified to not carry the plasmid. Strains with intermediate scores (pictured here in green, score 1 to 999) were removed from the analysis.

Of the 4433 strains originally included in the analysis, 3305 genomes met the inclusion criteria of 100% coverage of the 10 target sequences or 0% coverage with those in the intermediate range removed from the analysis (Figure 1). From there, after a preliminary tree was constructed, 29 additional genomes were removed; five that were determined to be a distant outgroup and 24 that were identified to not be *Salmonella* Infantis (none of the 29 strains contained the pESI plasmid). The analysis was conducted on 3276 *S*. Infantis strains, 1993 that had the pESI plasmid and 1283 that did not (Supplemental Table S1).

### 3.2. Metadata Summary

**Figure 2:**
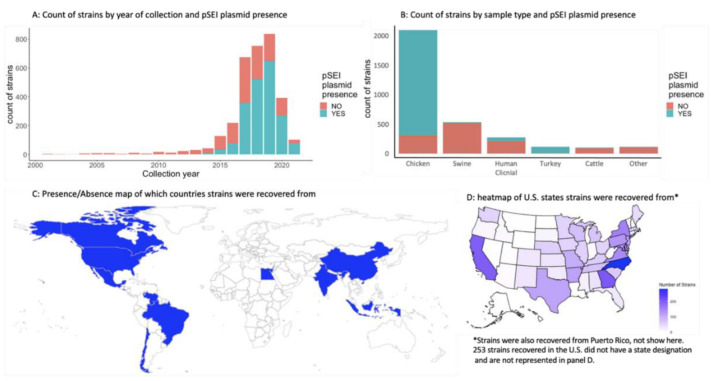
Metadata summary of *Salmonella* Infantis strains included in the study by (**A**) year of collection, (**B**) environment of isolation, and (**C**,**D**) geographical location of collection.

The 3276 strains included in the study were from the CDC (*n* = 260), FDA (*n* = 449), and FSIS (*n* = 2567); each group contained strains both with and without the pESI plasmid. Strains were collected over twenty years, from 2001 to 2021, with a higher proportion of strains carrying the plasmid collected after 2016 (Figure 2A). Two hundred seventy-seven isolates were collected from a clinical setting, whereas the remaining were recovered from various animal-associated raw meat sources, with the majority of these originating from chicken, swine, turkey, and cattle (Figure 2B). The strains fell into 114 clusters in the NCBI’s Pathogen Detection database (PDD) with 44 isolates (four with the plasmid 40 without) not associated with a cluster. Those strains without the plasmid were distributed within 111 clusters (the top 10 clusters contained 67% of strains without plasmids), whereas the cluster distribution of those with the plasmid was much narrower. All isolates carrying the plasmid fell into four clusters, and of those, 99.5% within just one cluster: PDS000089910.106. Those that did not fall into that cluster had no common metadata characteristics; two were U.S. clinical samples and three were environmental isolates from Egypt, Israel, and the United States. Strains were isolated from 12 countries, and at least 47 states and territories in the U.S. (Figure 2C,D); 253 strains were associated with the U.S. but did not have a state designation.

### 3.3. Description of the Population

**Figure 3:**
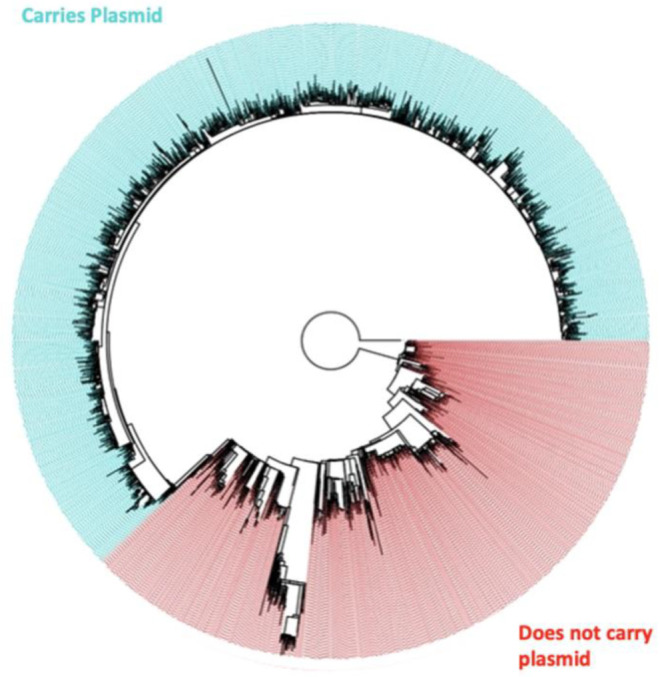
Phylogenetic tree of all *Salmonella* Infantis strains included in the analysis colored by plasmid carriage.

A phylogenetic tree encompassing all genomes was constructed and clear clades were observed between chromosomes that carried the pESI plasmid and those that did not (Figure 3). These differences were further examined through SNP, pangenome, and insertion sequence differences between the two groups.

### 3.4. SNP Differences

**Figure 4:**
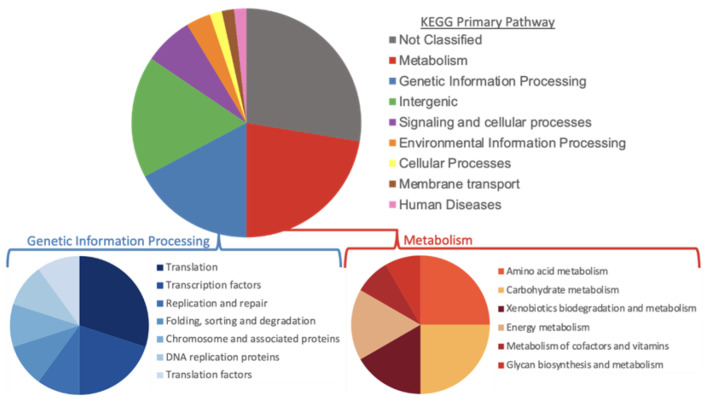
Overview of KEGG pathway classification of the 58 SNPs of interest that differ between *Salmonella* Infantis pESI plasmid carriage and those that do not carry the plasmid.

Using parsnp to determine core-genome informative SNPs, 21,737 total SNPs were included; of these, 15,206 were singletons and 20,587 occurred in less than 10 strains (<0.05% of the population). When differences between chromosomes that did and did not have the plasmid were described, 58 SNP were found in 100% linkage disequilibrium (LD) between groups in 3262 of 3305 genomes investigated (Supplemental Table S2). When the genes carrying these SNPs were grouped by higher-level functionality, 28% were unable to be classified and 17% were intergenic; of the classified genes, metabolism and genetic information and processing were the two most common pathways (Figure 4). Additionally, 14 strains (10 without the plasmid and 4 with the plasmid) did differ from the 58 SNP patterns at 22 nucleotide locations within the chromosome. Within the plasmid carriage group there were two groups, each containing two strains, that differed from the expected LD pattern. In the group without the pESI plasmid, there were three strains with unique differences not replicated in other strains and one group of seven strains that all had 19 SNPs in LD that were not expected.

### 3.5. Pan-Genome Analysis

In total, 4093 genes made up the core genome of all chromosomes, whereas 12,325 genes made up the pan-genome (not including the 324 genes associated with the plasmid). There were no genes found within just one plasmid carriage group that met the 99/1% or 95/5% cutoffs. Nineteen genes were found in more than half of the non-plasmid carriage group and not in any of the strains that harbored a plasmid and included hypothetical proteins, prophage integrase *IntS*, protein *UmuD*, putative defective protein *IntQ*, and SOS response-associated protein *YedK*. When only those genes present on the chromosome of isolates containing the plasmid were considered in a similar way, there were no genes unique to this strain. 

### 3.6. Insertion Element Family Differences

**Figure 5:**
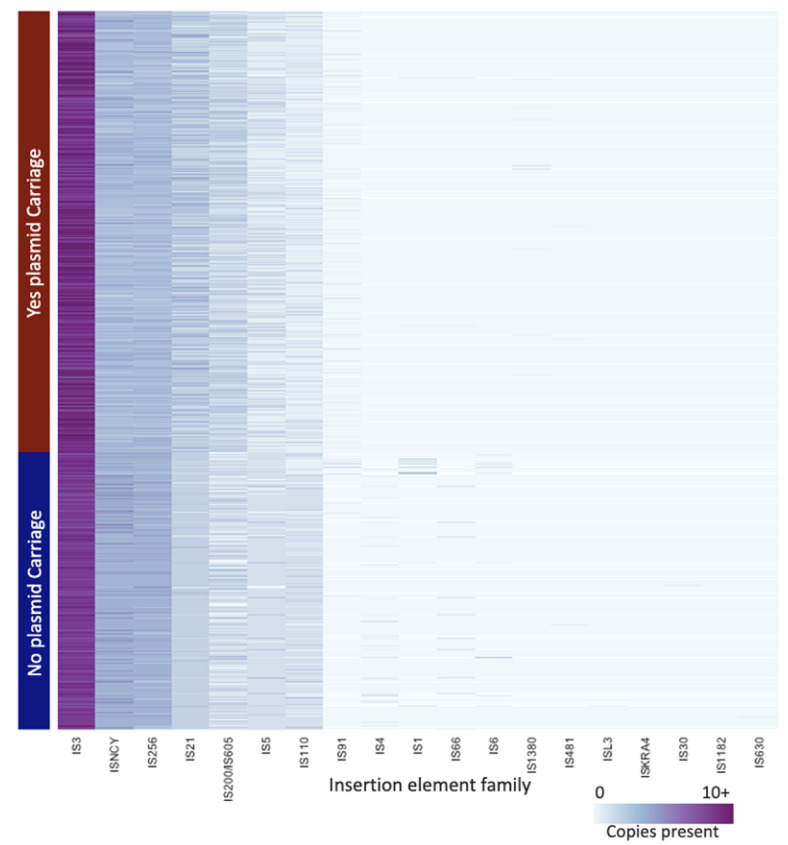
Heat map of number of insertion elements present in each chromosome. Each row represents a different isolate and is grouped by if the chromosome carries the pESI plasmid; each column is a different insertion element family. The darker the cell the higher number of insertion elements from that family are present in that strain.

When insertion elements in the chromosome were considered, 19 families were identified (Figure 5). On average, chromosomes from isolates that carried the plasmid had a higher (*p <* 0.05) number of insertion elements than chromosomes from isolates that did not carry the plasmid (32.9 total versus 29.3, respectively (SEM = 0.11)). Across chromosomes from isolates that carried and did not carry the plasmid, the IS3 family occurred at the highest level, followed by ISNCY and IS256. 

### 3.7. Describing the Intermediate Groups

Within the dataset, the 14 strains that differed from the 58 SNPs in LD in the SNP analysis were examined more closely. Only chromosomes that were not singletons in their SNP patterns were considered; this resulted in two groups with pESI plasmid carriage (GP + 1 and GP + 2) and one group without (GP-1). Each of the two groups with pESI plasmid carriage that differed both comprised two strains, GP + 1 from one U.S. clinical sample and an Israeli basil sample and GP + 2 from a U.S. clinical trial and Egyptian lemongrass tea. Both of these groups that carried the plasmid had one unique SNP and a common SNP across groups in an intergenic region (Table 1). When the group without a plasmid that did not follow common LD was examined, it had 19 unexpected SNPs given the lack of plasmid carriage; six in intergenic regions and 13 within genes (Table 1). Interestingly, all the strains in this group were associated with swine production (meat product or sows) in Wisconsin (*n* = 5) and New York (*n* = 1) within a four-year span.

## 4. Discussion

*Salmonella* Infantis has become a prominent serotype associated with U.S. raw chicken and turkey meat and increased human illnesses, highlighting the need to understand the factors allowing this serotype to proliferate in poultry-associated environments. Several studies have established the potentially advantageous effects of genes on the pESI plasmid in a poultry host [8,9]. In the present study, we investigated the chromosome of over 3200 *Salmonella* Infantis strains collected by the USDA-FSIS, CDC, and FDA and showed that clonal expansion of a strain carrying the pESI plasmid is responsible for the increased prevalence of serotype Infantis in samples of U.S. raw chicken and turkey meat.

Several genes on the pESI plasmid have been demonstrated to convey a possible advantage in poultry. For example, the fimbriae genes present have been shown to allow for better attachment to chicken and human epithelial cells in vitro and the iron siderophore system could provide an advantage in an iron-limited environment, such as a chicken host [8,20,21]. In contrast to genes found on the plasmid, the data suggest that the chromosome of the *S*. Infantis strains carrying the pESI plasmid do not specifically encode any special genes related to persistence in poultry or increased human infections, as there were no genes found to be unique to the chromosome in isolates carrying the plasmid. 

It is possible however, that genes present in strains both with and without the plasmid could influence persistence in a poultry host if mutated in those carrying the plasmid. Fifty-eight SNPs were in complete LD among strains carrying the pESI plasmid, but further investigation of phenotypic characteristics would be required to determine if any of these SNPs convey an advantage in a poultry host versus strains without the SNPs. Although LD can be caused by many evolutionary processes, including selection sweep resulting in hitchhiking genes, it is likely some of these SNPs are biologically significant between the groups. For example, one of the SNPs in LD was in the *gyr*A gene, resulting in an amino acid change, D87Y, that conveys resistance to fluoroquinolone antibiotics, which are a primary choice for salmonellosis treatment. It was previously determined that *S*. Infantis isolates carrying the pESI plasmid were resistant to fluoroquinolone antibiotics and that the resistance was not carried on the pESI plasmid [5,6]. The SNP in the multidrug efflux pump gene *acr*D could also be influencing antimicrobial susceptibility of the strain, but would need to be confirmed phenotypically [22].

In addition, several genes related to metabolism contained SNPs that could possibly influence persistence in a poultry environment. Chicken and turkey hosts can be nutrient-limited environments for essential bacterial growth factors, such as iron, and mutations that allow for increased acquisition, more efficient usage, or alternative energy sources would give this strain of *S*. Infantis a growth advantage over other *Salmonella* which do not contain the mutation, as well as other bacteria without these systems [23]. *cob*S involved in cobalamine synthesis, *eut*E involved in ethanolamine usage, *cys*P involved in thiosulfate usage, and *pdu*P involved in propanediol degradation all contained SNPs [24,25,26,27]. These systems are encoded by nearly 1% of the genome and, together, they enable *Salmonella* to use carbon sources and a terminal electron acceptor enabling anaerobic respiration unique to *Salmonella*. This advantage allows *Salmonella* to outgrow competing bacteria in the lumen of the intestine and be transmitted to new hosts via excretion. If these SNPs allowed for more efficient metabolism of these energy sources, this *S*. Infantis strain could have a metabolic advantage over strains without the SNPs, as well as advantages over other competing bacteria.

Additionally, there were SNPs in two signaling pathway genes: *fim*F and *bss*S. The *fim*F gene encodes the adaptor for type I fimbriae and is essential for fimbrial formation in *Salmonella* [28]. Changes in fimbrial adaptors have been shown to allow for structural changes in the assembled fimbriae [29]. If the SNPs present caused a change allowing for stronger attachment to epithelial cells, the strain possessing the gene could have an advantage over other bacteria without the increased attachment capabilities. The *bss*S gene is involved in regulation of biofilm formation in *Escherichia coli*, but its role has not been confirmed in *Salmonella*. However, *bss*S has shown to be upregulated during chlorine oxidation in *Salmonella* [30]. Chlorine compounds have been used in poultry processing for reduction of *Salmonella* and other pathogens.

In some bacteria, such as *Mycobacterium tuberculosis* and *Escherichia coli* 0157, insertion sequences can be used to further classify strains into specific types [31,32]. Although the presented results do not support this strategy for *S*. Infantis, the increased presence of IS elements can be indicative of different evolutionary mechanisms. IS expansion, the presence of many insertion sequences in a genome, can be the first step of genomic streamlining where a bacterium can lose gene functionality and become host dependent [33]. However, further investigation would be needed to determine what, if anything, the higher prevalence of IS elements indicates for this strain.

The results presented here agree with the results of both Tyson et al., 2021 and Gyomese et al., 2020: that a single clone containing the pESI is proliferating in poultry-related environments and causing an increase in human illnesses [10,11]. These data build upon Tyson’s work by defining the specific SNP differences in the chromosome beyond reporting clade membership. Here, we define the Infantis clone carrying pESI as containing 58 SNPs in LD between *S*. Infantis strains with and without pESI, further defining the 50 SNP difference group used by the NCBI’s Pathogen Detection database. 

Although greater than 99% of whole genomes containing the plasmid were members of a single clade, there were a few isolates from imported foods and clinical cases that did not align with the clade. This could indicate another strain carrying pESI circulating internationally that is different from the clone circulating in the U.S. Other studies have hypothesized that the pESI plasmid emerged in South America before identification in the U. S. The results presented here support the hypothesis that the *S*. Infantis strain acquired pESI and the strain was introduced to the U.S. where it then spread rapidly, rather than multiple strains acquiring the plasmid and spreading throughout the U.S.

*S*. Infantis has been consistently among the commonly isolated serotypes from raw poultry samples collected in processing facilities and in retail stores for the past five years [3,4]. Prior to 2016, isolation rates ranged between 4 and 10% [34]. Only after the emergence of the strain containing the pESI plasmid did the rates of isolation increase to over 30% in 2021 [4]. However, other strains not containing the plasmid are still isolated at rates similar to those prior to the emergence of the plasmid [9]. Combined with the results of the chromosomal genetic analysis and the plasmid genetic analysis, the conclusion can be reached that the proliferation of serotype Infantis in U. S. poultry is likely related more to the presence of the pESI plasmid than factors specific to Infantis as a serotype.

## Figures and Tables

**Table 1:** Overview of single-nucleotide polymorphism differences in intermediate *Salmonella* Infantis groups.

Chromosome Position	Gene Function	Ref. Allele (pESI+) *	Alt. Allele (pESI-)	Strain Groups	
Intermediate groups that do not carry the pESI-like plasmid				GP-1 ^1^ (*n* = 7)	
944796	Intergenic	A	C	A	
953613	AraC family transcriptional regulator	A	G	A	
1146423	Intergenic	A	C	A	
1176706	NAD(P)H nitroreductase	A	G	A	
1512725	Nitroreductase A	A	G	A	
1626589	Cell division protein ZapC	T	G	T	
1771815	NADH dehydrogenase	A	G	A	
2361903	Intergenic	C	T	C	
2451383	Carbon-nitrogen hydrolase	A	T	A	
2485165	Penicillin-binding protein 2	A	G	A	
2552207	Intergenic	C	T	C	
2577135	*cob*S	A	T	A	
3052001	PTS sugar transporter	T	C	T	
3373891	Alanine-tRNA ligase	T	C	T	
3557602	Exodeoxyribonuclease V subunit gamma	G	T	G	
3694291	ATPase	T	C	T	
3883819	Intergenic	A	C	A	
4385282	Intergenic	A	C	A	
4522651	ATP-dependent protease	C	G	C	
Intermediate groups that do carry the pESI-like plasmid				GP + 1 ^2^ (*n* = 2)	GP + 2 ^3^ (*n* = 2)
1774378	Peptidoglycan-binding protein LysM	G	A	G	A
2836180	DNA gyrase subunit A	A	C	C	A
4137324	Intergenic	A	T	T	T

* pESI-like plasmid. ^1^ Group GP-1 contains strains: PDT000300014.2, PDT000570019.1, PDT000336208.1, PDT000162382.2, PDT000159010.2, PDT000315325.2, and PDT000259666.2. ^2^ Group GP + 1 contains strains: PDS000032399.8 and PDS000032399.8. ^3^ Group GP + 2 contains strains: PDS000003946.16 and PDS000003946.16.

## Data Availability

Sequences used in this study are publicly available and can be downloaded via the NCBI’s Isolates Browser of the Pathogen Detection database.

## Supplementary Materials

The following supporting information can be downloaded at: https://www.mdpi.com/article/10.3390/microorganisms10071478/s1, Table S1: Metadata of isolates included in the study. Table S2: 58 SNPs of interest only present in isolates carrying pESI.
